# Musical Training and Brain Volume in Older Adults

**DOI:** 10.3390/brainsci11010050

**Published:** 2021-01-05

**Authors:** Laura Chaddock-Heyman, Psyche Loui, Timothy B. Weng, Robert Weisshappel, Edward McAuley, Arthur F. Kramer

**Affiliations:** 1 Beckman Institute, University of Illinois at Urbana-Champaign, Urbana, IL 61801, USA; rawrbert88@gmail.com (R.W.); emcauley@illinois.edu (E.M.); afkramer@illinois.edu (A.F.K.); 2 Department of Psychology, Northeastern University, Boston, MA 02115, USA; p.loui@northeastern.edu; 3 Department of Diagnostic Medicine, The University of Texas at Austin, Austin, TX 78712, USA; Timothy.Weng@austin.utexas.edu; 4 Department of Kinesiology and Community Health, University of Illinois at Urbana-Champaign, Urbana, IL 61801, USA

**Keywords:** music, aging, brain structure, musical training, older adults

## Abstract

Musical practice, including musical training and musical performance, has been found to benefit cognitive function in older adults. Less is known about the role of musical experiences on brain structure in older adults. The present study examined the role of different types of musical behaviors on brain structure in older adults. We administered the Goldsmiths Musical Sophistication Index, a questionnaire that includes questions about a variety of musical behaviors, including performance on an instrument, musical practice, allocation of time to music, musical listening expertise, and emotional responses to music. We demonstrated that musical training, defined as the extent of musical training, musical practice, and musicianship, was positively and significantly associated with the volume of the inferior frontal cortex and parahippocampus. In addition, musical training was positively associated with volume of the posterior cingulate cortex, insula, and medial orbitofrontal cortex. Together, the present study suggests that musical behaviors relate to a circuit of brain regions involved in executive function, memory, language, and emotion. As gray matter often declines with age, our study has promising implications for the positive role of musical practice on aging brain health.

## 1. Introduction

Aging is accompanied by changes in cognition and brain health [1,2,3,4,5]. In general, older adults show declines in performance on cognitive tasks of executive function and memory, and performance decreases are accompanied by decreases in gray and white matter volume [6,7,8,9]. However, there are individual differences and variability in the trajectory of changes in cognition and brain health with aging. Some individuals maintain cognitive skills and brain structure, while others exhibit decline [10].

Indeed, considerable evidence suggests that the brains of older adults have the capacity for plasticity [11]. For example, engagement in physical activity (e.g., aerobic exercise [12,13,14]; non-aerobic exercise [15]; ballet [16]) and cognitively stimulating leisure activities (e.g., music [17], dancing [18], and chess [19]) have been shown to benefit the aging brain and cognition, as well as to reduce the risk of dementia [20]. Given the increasing aging population and the expected rise in the prevalence of Alzheimer’s disease over the next 40 years [21], it is important to determine lifestyle factors that benefit the aging brain.

Musical practice has been found to benefit cognitive function and brain health in older adults [17]. Musical training involves sensory systems, motor systems, and cognitive processes at multiple levels [22,23], and playing a musical instrument is associated with superior cognitive performance in older adults [17,24,25]. Interestingly, the cognitive benefits of music in older adults extend beyond tasks related to musical skills to include cognitive processes such as executive function, attention, inhibition, memory, cognitive flexibility, spatial ability, and processing speed [17]. Variables relating to musical practice, such as the intensity of the musical activity [26], maintaining the practice into older age [26,27,28], type of training [26], training duration [28], and earlier age of onset [26,27,29] have also been found to play a role in cognitive sparing and improvements.

Less is known about the role of musical practice and musical experiences on brain health, particularly in older adults. In terms of brain structure, young adult musicians and non-musicians (aged 18–40 years) have been found to differ in brain structure in areas involved in perceptual, motor, auditory, somatosensory, and cognitive functions [30,31]. Specifically, one study demonstrated that young adult professional keyboard players and amateurs/non-musicians showed differences in gray matter volume of motor, auditory, and visual-spatial brain regions [30]. Another study examined gray matter density (via voxel-based morphometry, or VBM) in young adults (age 25) with three distinct, progressive levels of musical training intensity/expertise [31]. Gray matter density increased with expertise in musical training in frontal and parietal brain areas (e.g., inferior frontal gyrus—regions said to be important for executive function, working memory, and syntactic processing), mid-orbital gyrus (involved in tonal sensitivity), and intraparietal sulcus (with a role in visual–motor coordination), among other brain regions [31]. Conversely, gray matter density decreased with expertise in musical training in the striatum, which may reflect automation of motor abilities with music [31]. Specific musical abilities have been found to play a role in gray matter volume as well: a voxel-based morphometry study showed that gray matter volume in the cerebellum was associated with beat discrimination abilities in the general population [32], with expert dancers showing especially lower gray matter density in the cerebellum [33]. To our knowledge, little is known about the role of music training on gray matter structure in older adults.

The present study explored the role of different types of musical behaviors on brain structure in older adults. In an older adult cohort, we were interested in the relationship between self-report measures of musical training and musical appreciation and structural brain measures. We administered the Goldsmiths Musical Sophistication Index (Gold-MSI v1.0, 11 October 2012), a questionnaire that includes questions about a variety of musical behaviors, including performance on an instrument, musical practice, allocation of time to music, musical listening expertise, and emotional responses to music [34]. The Gold-MSI has emerged in recent years as a frequently used, well-validated, self-report inventory that captures several aspects of musical experience, including musical training and appreciation. In fact, the Gold-MSI self-report scale has been validated in a large sample across the lifespan (n of 147,636 in a broad age range as of 2014: [34]). The Gold-MSI is a broad index of multi-faceted musical behaviors designed to conceptualize the specific nature of musical expertise across individuals of all musical backgrounds. In general, higher levels of musical sophistication are characterized by higher frequencies of musical behaviors, greater accuracy or ease when involved in musical behaviors, and a varied repertoire of musical behaviors [34]. We hypothesized that older adults with higher levels of musical sophistication would show greater brain volumes in regions across the cortex. Nevertheless, we examined both positive and negative associations between musical training and brain structure (see [31,35]). Based on other aging and lifestyle studies, we predicted associations between musical sophistication and brain structure in regions important for executive function and memory.

## 2. Method

### 2.1. Participants

Healthy older adults (aged 60–80) were recruited from the Urbana-Champaign community. Selection criteria consisted of the following: (1) >75% right-handed on the Edinburgh Handedness Questionnaire; (2) normal or corrected-to-normal vision of at least 20/40; (3) no color-blindness; (4) no history of stroke, transient ischemic attack, or head trauma; (5) >23 score on the Mini-Mental State Examination (MMSE); (6) >21 score on the Telephone Interview of Cognitive Status (TICS); (7) <10 score on the Geriatric Depression Scale (GDS); (8) self-report of no more than 30+ minutes of moderate-intensity exercise twice per week in the last 6 months (given recruitment for an exercise intervention); and (9) safely able to participate in an MRI environment (e.g., no claustrophobia or metallic implants).

The participants were originally recruited and scanned in the MRI for a randomized controlled exercise trial (https://clinicaltrials.gov/ct2/show/NCT01472744). The MRI data in the present study corresponded to the pre-intervention MRI, which occurred between 5 and 8 years (mean = 6.5 years) before the collection of the music survey data about lifetime musical experiences. We sent a letter via mail to the participants who had previously completed the exercise study to ask whether they were interested in completing a short questionnaire about their lifetime musical experiences for USD 10 compensation. All participants provided informed consent, and the University of Illinois Institutional Review Board approved all procedures used in the study (protocol number: 19776).

**Figure 1:**
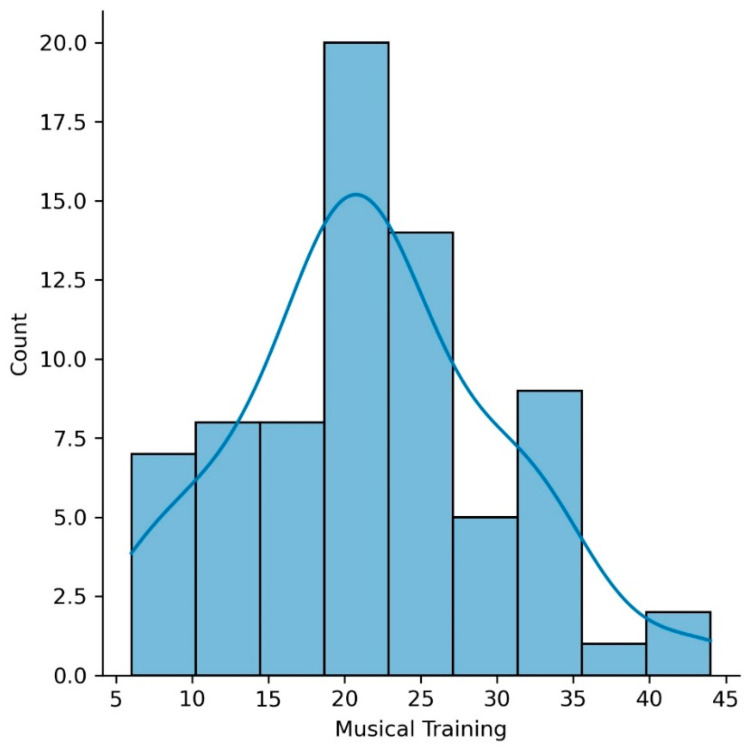
Histogram of the Musical Training Goldsmiths Musical Sophistication Index (Gold-MSI) scores for our sample.

Seventy-three older adults completed the music questionnaire (mean age of 65.9 years (SD = 4.5); mean education of 16.2 years (SD = 2.8)). Our sample included participants with a variety of musical experiences, including amateur musicians with high, intermediate, and low levels of musical practice, as well as non-musicians. None of the participants reported being professional musicians. Figure 1 shows a histogram of the Musical Training Gold-MSI scores for our sample.

### 2.2. Music Questionnaire

We administered the Goldsmiths Musical Sophistication Index, v1.0, 11 October 2012 (https://www.gold.ac.uk/media/documents-by-section/departments/psychology/full_gmsi-1.pdf). The questionnaire includes questions about a variety of musical behaviors, including performance on an instrument, listening expertise, communication about music, etc. [34].

While previous studies that investigated the cognitive and brain differences between musicians and non-musicians have defined musicians as individuals with formal musical training (e.g., instrument, voice), these studies do not account for musical expertise that does not involve technical musical knowledge. Specifically, knowledge from musical listening and appreciation may be implicitly acquired even in those without formal musical training. The musical sophistication index is a broad index of multi-faceted musical behaviors aimed to conceptualize the specific nature of musical expertise across individuals of all musical backgrounds. “High levels of musical sophistication are said to be characterized by (a) higher frequencies of exerting musical skills or behaviors, (b) greater ease, accuracy, or effect of musical behaviors when executed, and (c) a greater and more varied repertoire of musical behavior patterns. This means that highly musically sophisticated individuals are able to respond to a greater range of musical situations, are more flexible in their responses, and possess more effective means of achieving their goals when engaging with music” [34].

Responses were entered into the Gold-MSI scoring template for scoring (https://www.gold.ac.uk/media/documents-by-section/departments/psychology/Gold-MSIv10_All_Items_Scoring_Template.xls).

The Goldsmiths Musical Sophistication Index classifies musical skills and behaviors into five factors/dimensions. See Table 1 for the composite scores of the five Gold-MSI subscales for the present study and the percentile scores relative to the data norms of the Gold-MSI [34].

### 2.3. Active Engagement

- Twenty items;
- Active musical engagement behaviors (e.g., “I keep track of new music that I come across”, “I often read or search the internet for things related to music”);
- Deliberate allocation of time and money on musical activities (e.g., “I don’t spend much of my disposable income on music”, “I listen attentively to music for _ hours per day”).

### 2.4. Perceptual Abilities

- Fifteen items;
- Self-assessment of a cognitive musical ability, most of them related to musical listening skills;
- Music listening skills (e.g., “I can compare and discuss differences between two performances or versions of a musical piece”, “I can tell when people sing or play out of tune”).

### 2.5. Musical Training

- Eleven items;
- Extent of musical training and practice (e.g., “I engaged in regular daily practice of a musical instrument including voice for __ years”, “At the peak of my interest I practiced on my primary instrument including voice for __ hours per day”);
- Degree of self-assessed musicianship (“I would not consider myself a musician”, “I have never been complimented for my talents as a musical performer”).

### 2.6. Singing Abilities

- Seven items
- Skills and activities related to singing (e.g., “After hearing a new song two or three times I can usually sing it by myself”, “I am not able to sing in harmony when somebody is singing a familiar tune”).

### 2.7. Emotions

- Nine items;
- Mainly active behaviors related to emotional responses to music (e.g., “I am able to talk about the emotions that a piece of music evokes in me”, “I sometimes choose music that can trigger shivers down my spine”).

### 2.8. Brain Structure

For all participants, high-resolution T1-weighted structural brain images were acquired before the randomized controlled exercise trial using a 3D MPRAGE (Magnetization Prepared Rapid Gradient Echo Imaging) protocol with the following parameters: GRAPPA acceleration factor 2, voxel size = 0.9 × 0.9 × 0.9 mm, repetition time (TR) = 1900 ms, TI = 900 ms, TE = 2.32 ms, flip angle = 9°, FoV = 230 mm. All images were collected on a 3-T head-only Siemens Allegra MRI scanner.

Automated brain tissue segmentation and reconstruction of cortical surface models were performed on T1-weighted structural MRI images using the standard recon-all image processing pipeline in FreeSurfer version 5.3 (http://surfer-nmr.mgh.harvard.edu/). FreeSurfer automatically labels cortical surfaces using a Desikan–Killiany cortical parcellation atlas (see [36] for the labeling protocol). That is, vertices along the cortical surface are assigned a given label based on local surface curvature, average convexity, prior label probabilities, and neighboring vertex labels [36,37]. Data from all participants were processed using the same Apple OSX computer to ensure that the observed findings were not a function of differences in software, operating system, or hardware specifications [38].

Specifically, the following processing stream was applied to each participant’s structural image via FreeSurfer’s recon-all processing pipeline: (1) non-brain tissue removal, (2) Talairach transformation, (3) creation of representations of the gray/white matter boundaries [39,40], and (4) calculation of the cortical thickness as the distance between the gray/white matter boundary and the pial surface in all regions of interest [41]. Talairach transforms, skull stripping, gray–white tissue segmentation, and surface reconstructions were visually checked for errors.

Regions of interest available via FreeSurfer cortical parcellations include frontal cortex (superior, middle, inferior, orbitofrontal), parietal cortex (superior, inferior, posterior cingulate), temporal cortex (superior, inferior, middle, parahippocampal), and insula, as offered in FreeSurfer’s segmentation algorithms [42]. These areas provide an exploratory analysis of the whole brain. Brain volume was measured in cubic millimeters. Our main outcome was total brain volume (sum of left and right hemispheres). We first aggregated the brain volume measures across left and right hemispheres to reduce the number of statistical comparisons in relating brain volume to behavior. Then, for lateralized (not midline) regions that were found to be significantly associated with musical behaviors, we further explored the laterality of the results by separately correlating the brain volume of specific regions in the left and right hemispheres with the musical measures.

### 2.9. Statistical Analysis

Correlations between composite scores corresponding to each musical behavior (Active Engagement, Perceptual Abilities, Musical Training, Singing Abilities, Emotions; Table 1) and demographic variables (age, sex, years of education, MMSE) were conducted to determine relevant covariates.

Linear regressions were employed to test associations between musical behaviors and brain volume, controlling for intracranial volume (ICV). T-scores and standardized betas (β) are presented. Benjamini–Hochberg correction for multiple comparisons was applied with the false-discovery rate set at *p* = 0.05 to show significant associations after correcting for the 12 regions of interest across the brain. We also explored associations at the *p* < 0.05, uncorrected level. While there may be some false positives in the uncorrected (*p* < 0.05) results, we present the uncorrected associations as additional exploratory analyses that remain to be tested further in future studies with larger sample sizes.

## 3. Results

### 3.1. Music, Age, Sex, and Education

Musical Training was not significantly associated with age, sex, or years of education. Perceptual Abilities were inversely correlated to age (*r* = −0.255, *p* = 0.028) and positively associated with education (*r* = 0.268, *p* = 0.021) and MMSE (*r* = 0.294, *p* = 0.011). Active Engagement, Singing, and Emotions were not significantly associated with age, sex, or education. Emotions were positively associated with MMSE (*r* = 0.258, *p* = 0.027).

### 3.2. Musical Training and Bilateral Brain Volume

**Figure 2:**
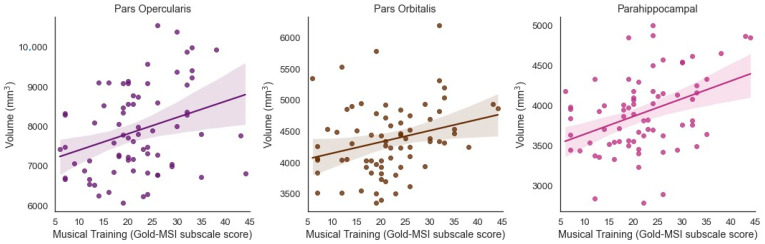
Significant positive associations between Musical Training (Gold-MSI) and volume of the bilateral inferior frontal cortex (pars opercularis and pars orbitalis) and parahippocampus in older adults (Benjamini-Hochberg corrected).

Musical Training was significantly and positively associated with the volume of the bilateral inferior frontal cortex pars opercularis (β = 0.314, *t* = 3.035, *p* = 0.003, adjusted *p* = 0.0195) and bilateral pars orbitalis (β = 0.268, *t* = 2.749, *p* = 0.008, adjusted *p* = 0.0347) when controlling for ICV (Figure 2). Musical Training was significantly and positively associated with the volume of the bilateral parahippocampus (β = 0.390, *t* = 3.588, *p* = 0.001, adjusted *p* = 0.013) when controlling for ICV (Figure 2).

### 3.3. Laterality of Results

For the above regions, we further examined the laterality of associations between musical training and brain volume by separately correlating left and right hemisphere regions with the music survey scores. In correcting for the unilateral brain regions that showed associations between Musical Training and bilateral brain volume (inferior frontal cortex pars orbitalis, inferior frontal cortex pars opercularis, parahippocampus), we applied Bonferroni correction for multiple comparisons, resulting in a corrected threshold *p*-value of 0.0083 (*p* = 0.05/6 unilateral regions = 0.0083).

**Figure 3:**
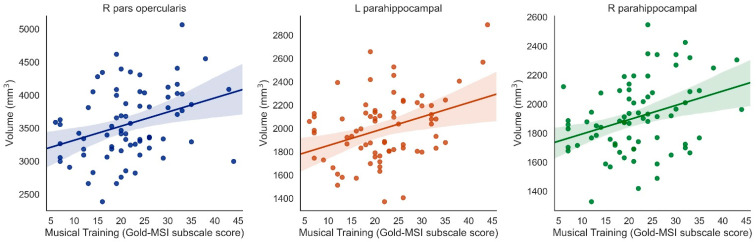
Significant positive associations between Musical Training (Gold-MSI) and right pars opercularis, left parahippocampus, and right parahippocampus (Bonferroni-corrected).

Musical Training was positively associated with right pars opercularis (β = 0.338, *t* = 3.389, *p* = 0.001, surviving correction for multiple comparisons), left pars opercularis (β = 0.224, *t* = 2.000, *p* = 0.049, uncorrected only), left pars orbitalis (β = 0.253, *t* = 2.577, *p* = 0.01, uncorrected only), right pars orbitalis (β = 0.222. *t* = 2.084, *p* = 0.041, uncorrected only), and both left and right parahippocampus (left: β = 0.364, *t* = 3.277, *p* = 0.002; right: β = 0.338, *t* = 3.069, *p* = 0.003, both surviving correction for multiple comparisons) (when controlling for ICV) (Figure 3).

### 3.4. Exploratory Analyses (Uncorrected, p < 0.05)

**Figure 4:**
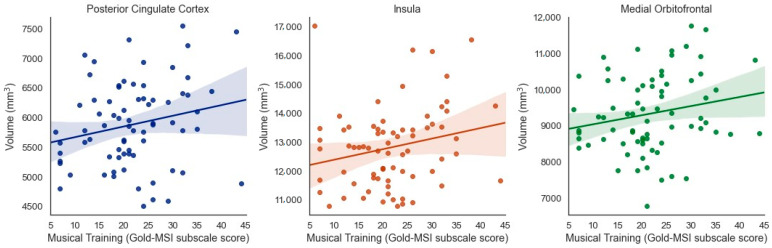
Positive associations between Musical Training (Gold-MSI) and volume of the bilateral posterior cingulate cortex, insula, and medial orbitofrontal cortex in older adults (uncorrected).

When considering results that were significant at the uncorrected level but did not pass correction for multiple comparisons, several additional (bilateral) regions emerged as being potentially associated with specific aspects of musical experience. Musical Training was positively associated with brain volume of the posterior cingulate cortex (β = 0.211, *t* = 2.020, *p* = 0.047, adjusted *p* = 0.07), insula (β = 0.219 *t* = 2.028, *p* = 0.046, adjusted *p* = 0.07), and medial orbitofrontal cortex (β = 0.195, *t* = 2.049, *p* = 0.044, adjusted *p* = 0.07) when controlling for ICV (Figure 4).

**Figure 5:**
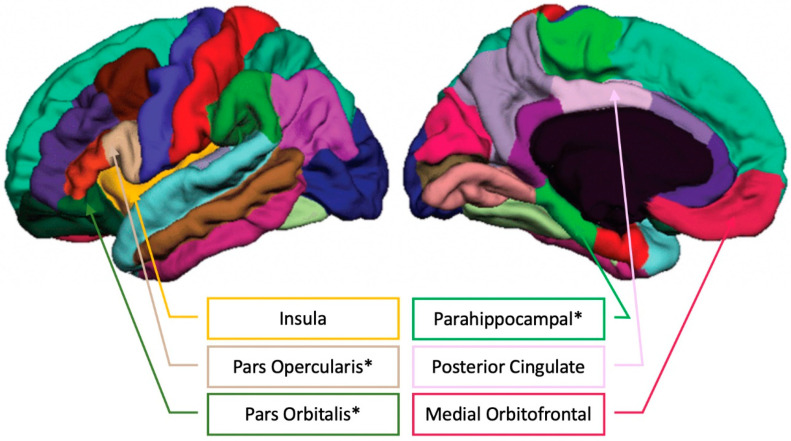
FreeSurfer cortical parcellations with the regions showing positive correlations between Musical Training (Gold-MSI) and brain volume in older adults (please see [42] for the unlabeled FreeSurfer cortical parcellation image). * indicates passing the Benjamini–Hochberg correction for multiple comparisons.

See Figure 5 for an illustration of the FreeSurfer cortical parcellations positively related to musical training.

### 3.5. Specificity of Results

There were no significant associations between Active Engagement (i.e., allocation of time and money on musical activities; tracking new music; attentive listening to music), Perceptual Abilities (self-assessment of a cognitive musical ability; musical listening skills), Singing Abilities, or Musical Emotions (emotional responses to music) and brain volume (*p* > 0.05). There were no associations between musical behaviors and volume of superior or middle frontal cortex, superior or inferior parietal cortex, or superior, inferior, or middle temporal cortex (*p* > 0.05). There were no significant negative associations.

## 4. Discussion

While previous longitudinal studies have shown that music can improve cognition, emotion, and well-being among older adults [24,25,43], relatively little is known about the neural structures that underlie musical training and experience in older individuals. The present study demonstrated that musical behaviors were associated with brain volume in older adults. Our sample included participants with a variety of musical training: while no participant reported being a professional musician, participants did include amateur musicians with high, intermediate, and low levels of musical practice, as well as those with no explicit musical training. Musical training, defined as the extent of musical training, musical practice, and musicianship, was positively and significantly associated with volume of the inferior frontal cortex (pars orbitalis and pars opercularis) and parahippocampus (when considering correction for multiple comparisons). In addition, musical training was potentially positively associated with volume of the posterior cingulate cortex, insula, and medial orbitofrontal cortex, although the latter results did not pass correction for multiple comparisons. We interpret the associations between musical behaviors and the volume of groups of brain regions by considering the role of the brain regions in specific networks that enable executive function, memory, language processing, and emotion. Music practice in old age may relate to the volume of these brain regions due to its influence on cognitive functions, as shown in previous studies (e.g., [24,25,43]). In this regard, the present study adds to these previous behavioral studies on music and aging by adding the convergent measure of brain volume. Furthermore, as gray matter often declines with age, our results have promising implications for the positive role of music on aging brain health.

### 4.1. Music and Language

We report an association between musical training and volume of the inferior frontal gyrus (pars opercularis and pars orbitalis) in older adults. The inferior frontal gyrus is known to be involved in the perception and production of language, which includes speech (acoustic communication), but also signed and written communications. Interestingly, the inferior frontal gyrus is also involved in musical tasks. Music and language have been found to share representations and resources across many studies [44,45]. Music is linked to auditory processing, as music performance involves listening and sound discrimination [46]. Improvements in auditory processing for musicians extend to the demands of language, specifically speech processing, including auditory attention and speech perception in noise [47,48,49].

Due to the overlap in processing demands between music and language, the effects of musical training on language abilities may be considered a form of near transfer because of the relatively similar contexts surrounding music and language [50]. While far transfer involves transferring skills to dissimilar contexts or tasks, near transfer involves using the same or similar set of skills for multiple contexts [50]. In this regard, the effects of musical training on language may be a form of near transfer because the production of music and language both require skills such as auditory–motor sequencing and sound perception and production. Here, the finding that music training correlates with brain volume in the inferior frontal gyrus, which is classically associated with speech and language functions, but has been more recently shown to be active in many aspects of music processing as well (see [51,52,53]), provides further support for the idea of overlap between brain processing mechanisms for music and language [54].

In support of the shared framework of music and language, the aging auditory system often experiences disruptions in neural timing, or neural timing delays, in response to speech, which leads to difficulty in understanding speech and encoding sounds [55]. Interestingly, older adults with musical training do not experience neural timing delays in response to speech. That is, it seems that the neural representation of temporal features is strengthened with musical training [56]. Some claim that musical training requires mapping sounds to meaning, which may help the auditory system interact with sound (including speech).

Musicians have shown increased gray matter volume (via voxel-based morphometry) in the pars opercularis, which may represent structural brain changes with auditory and motor skill acquisitions [57]. In addition, musicians have increased gray matter in Broca’s area in the inferior frontal gyrus [58]. Broca’s area is said to be involved in sight reading and motor sequences involved in musical performance as well as visuospatial and audiospatial localization [59]. Patients with lesions in the Broca’s area show abnormal electrophysiological responses to musical syntax manipulations [52], suggesting that the left inferior frontal gyrus is causally involved in music processing. Furthermore, in younger adults, gray matter density was shown to increase with musical expertise in the left inferior frontal gyrus in a cluster extending from the pars triangularis to the insula [31]. The findings by Sammler et al. [52] and James et al. [31] are broadly consistent with the present results in showing associations between pars orbitalis and musical training. Our results are also broadly consistent with Bermudez et al. [60], who found differences between musicians and non-musicians in the right inferior frontal gyrus (both pars triangularis and pars orbitalis) in voxel-based morphometry, as well as the right parahippocampus in cortical thickness data, which we will discuss below. It is important to note that most previous research focused on professional musicians and/or professional and amateur musicians, in contrast to the present study, which included older adult participants with a range of musical experiences and varied musical status.

### 4.2. Music, Memory, and Executive Function

Musical training was positively associated with volume of the (left and right) parahippocampal cortex in older adults. This could be interpreted in the light of the parahippocampal cortex’s known roles in memory and emotion. Musical performance involves memorizing a musical piece and recalling the piece during a concert [61], as well as improved rehearsal mechanisms. Indeed, musicians have been found to encode, manipulate, and retrieve information differently from non-musicians [62,63]. Further, the processing of musical syntax requires comprehension of musical structure as well as tracking short-term and long-term musical context [64]. Musicians show faster updating of auditory and visual working memory representations compared to non-musicians [65].

Moreover, the parahippocampus is said to link the default mode cortical network with the medial temporal lobe memory system. Specifically, the parahippocampus has been found to mediate resting state connectivity between the hippocampus and posterior cingulate cortex (the hub of the default mode network) [66]. Here, we also identified associations between musical training and the volume of the posterior cingulate cortex in older adults (uncorrected). From a brain network approach, the posterior cingulate cortex is considered a core hub of the default mode network [67,68]. The default mode network is a brain network known to facilitate creative ideation as well as executive function [67,68], both of which are important in musical training, especially in cases such as jazz improvisation [69].

Indeed, executive function, which includes cognitive processes of inhibition, attention, and flexibility, is associated with musical abilities, as described by the OPERA hypothesis [54]. High levels of executive function are needed for musicians to inhibit information during a performance (e.g., other melodies, the audience) [70] as well as to monitor and shift skills during a performance [71]. Musical activities also create attentional demands [54]. Musicians playing in a group require strong sensorimotor skills to synchronize personal musical performance with group performance. Audio-spatial localization is needed to hear musical cues from other instruments [58]. In addition, a musician must divide attention as he or she attends to the musical score and the body movements of the conductor and group. Sustained attention and vigilance are needed to perform over extended periods of time [72]. Music also involves visuospatial ability via rapid reading of musical scores as well as an analysis of note location on a staff [58].

A few musical studies have linked musical ability or experience to the posterior cingulate cortex. One study showed that musicians had a reorganized thalamic-cortical functional brain network, including auditory areas and the posterior cingulate cortex [73]. The authors suggest this circuit may be relevant to higher sensitivity to sound as well as the integration of mental imagery and sound, both of which are important for musical performance [73]. In addition, the posterior cingulate, a node within the default mode network, has been shown to be more tightly coupled with the right executive control network in individuals with experience in musical improvisation, with the posterior cingulate cortex specifically being significantly correlated with creative behavior both in music and in non-musical psychometric tests of divergent thinking [69]. These findings suggest that the posterior cingulate may be involved as a hub in large-scale network changes that come with specific musical training, which may encourage musical ideation and idea generation.

### 4.3. Music, Emotion, and Reward

Parahippocampal-dependent memory processes are also linked to emotion, with shared limbic brain structures and interactions between the parahippocampal cortex and frontal cortex. Music elicits strong emotional responses, which are linked to parahippocampal and limbic structures as well as to neurochemical systems that support reward, motivation, stress and arousal, immunity, and social affiliation [74,75,76]. Indeed, individuals report a variety of sensations linked to pleasure when listening to music, including chills and goosebumps [77]. These sensations are perceived as pleasurable [78,79], and are linked to changes in heart rate and skin conductance [80], as well as neural activity in reward and emotional regions of the brain, including the insula, striatum, and orbitofrontal cortex [81,82,83].

We also identified an (uncorrected) association between musical training and volume of the insula and medial orbitofrontal cortex in older adults. From a brain network approach, the insula is often considered the core of the salience network. The salience network is known to be important for sustained task-set maintenance, error feedback for tuning top-down control, and maintaining associations between actions and outcomes, all functions important for musical activities [84,85]. In particular, the salience network is said to first determine information consistent with behavioral goals by detecting and filtering salient stimuli. Then, the network helps to facilitate the involvement of attentional and working memory resources via recruitment of other large-scale brain networks [86,87] through the integration of sensory, emotional, and cognitive information [88]. Indeed, a musician must detect important information and then recruit attentional and memory resources for successful performance. In fact, some research suggests that the salience network helps modulate the switch between the internally directed default mode network and the externally directed executive network [89].

The insula is also important for expression of emotion. Recent work using resting state functional connectivity has shown that the insula is at the center of overlapping functional connectivity between areas in the auditory system (such as superior temporal areas) and areas in the dopaminergic reward system (striatum and medial prefrontal cortex), and that this insula-centered functional connectivity is preserved in older adults, even among older adults with Mild Cognitive Impairment and Alzheimer’s Disease [90]. Since the functional connectivity between auditory and the reward systems is crucial for strong emotional responses to music, this relationship between musical training and insula may suggest that the capacity for making connections between musical sounds and strong emotions is strengthened by musical training, an idea that is supported by associations between musical emotions and musical training in the general population [34].

White matter connectivity between emotional processing brain regions, including the insula and medial prefrontal cortex, and sensory processing brain areas (including the superior temporal gyrus) has been found to explain individual differences in reward sensitivity to music [91,92]. In addition, musicians have shown increased insular connectivity with regions involved in affective processing (e.g., orbitofrontal cortex), salience detection, and higher-order cognition, which may lead to faster integration of sensory information with musical performance [93]. Indeed, the orbitofrontal cortex is known for connections with sensory areas as well as dopaminergic reward-sensitive structures involved in emotion and memory. Cerebral blood flow has been found to increase in the orbitofrontal cortex as intensely pleasant emotional responses (e.g., chill intensity) increased [81], and white matter underlying the orbitofrontal cortex is associated with the frequency of chills experienced during music listening [92]. Furthermore, individuals who score highly on the Barcelona Music Reward Questionnaire, a measure for sensitivity to the rewards of music listening, have higher structural connectivity between white matter underlying the orbitofrontal cortex and right hemisphere auditory regions [94]. People with musical anhedonia (a specific lack of reward sensitivity to music) are deficient in brain connectivity between auditory and medial prefrontal cortex, again suggesting the role of this pattern of connectivity in enabling emotional responses to music [94]. Taken together, our findings suggest a relationship between musical training and the emotion and reward networks among older adults.

### 4.4. Limitations and Future Directions

The present study has important implications for the role of musical activities, particularly musical training, on brain structure in aging. As our study was cross-sectional, it will be interesting for future investigators to track changes in brain structure and function over time as a function of musical experiences. It will also be useful for future studies to collect self-reported musical activities and brain structure data at the same time. In the present study, we collected the musical self-report data several years after the conclusion of the study in which the MRI data were collected. Although it would be ideal to collect the MRI and musical self-report data in close temporal proximity, this is not critical, since the Gold-MSI questions focus on a lifetime of musical activities and practice.

The finding that musical training relates to brain volume in specific regions in an older adult sample may relate to the ongoing discussion on cognitive reserve. Cognitive reserve is the concept that engaging in enriching activities over the whole lifespan may be expressed in old age as resilience to neuropathological damage [95]. As music perception and production involves perceptual, motor, cognitive, and emotional functions, musical activities may be considered enriching activities. In that regard, a recent meta-analysis has linked cognitive reserve to music practice [17], with specific associations between onset of musical training and auditory or phonemic working memory [29,96]. The present results contribute brain volume data as a convergent method to add specificity to the relationships between musical training and previously observed behavioral outcomes.

Although the survey portion of the Gold-MSI provides a comprehensive subjective self-report assessment of multiple facets of musical experience, future studies may also employ behavioral tools to dive more deeply into specific aspects of musical experience, such as music performance, listening tests of absolute and relative pitch, rhythm discrimination and production, and memory, as well as emotional aspects of music perception and production, and relate these to gray and white matter in older adults. Future work may also consider incorporating global health, such as cardiovascular health, as additional covariates, as these are known to correlate with cognitive behavior, including motivated behaviors such as musical activity [97].

It will also be interesting for future work to explore the role of music on brain structure in individuals with dementia. Singing and musical listening have been found to improve cognitive performance and mood in older adults with mild–moderate dementia [98]. Furthermore, music interventions in older adults with Alzheimer’s Disease and Mild Cognitive Impairment have been shown to help preserve functional connectivity between auditory brain regions (e.g., temporal gyrus) and reward regions (e.g., basal ganglia, orbitofrontal cortex) at an early state of neurodegeneration [90]. As physical activity [12,13] and cognitive training [99,100] are also known to play a role in aging brain health and cognition, future researchers may explore the interactions among musical training, exercise training, and cognitive training to understand the role of each activity in the aging brain, as well as the best approaches to boosting brain and cognitive health in the elderly.

Finally, as illustrated in Table 1, our Gold-MSI scores were somewhat lower than the average scores in Müllensiefen et al. [34], especially on the Active Engagement subscale. This is likely because the present study only included older adults, whereas participants from Müllensiefen et al. included a broad age range. Müllensiefen et al. demonstrated that age was a significant predictor of scores on the Gold-MSI, with retired participants scoring significantly lower on musical sophistication. Thus, our results are consistent with previous reports in showing age-related effects of musical sophistication and musical engagement.

## 5. Conclusions

Our results arrive at an important time as the aging population increases [21]. The present study raises the possibility that musical training may help offset age-related declines in brain volume in older adults. We hope the results will encourage individuals to be involved in a lifetime of musical activities, particularly musical training, listening, and enjoyment. As mounting evidence supports the role of musical activities on brain health, the present results add specificity by relating multiple aspects of musical experience to regional gray matter in the aging brain [101].

## Figures and Tables

**Table 1:** Composite scores of the five Gold-MSI subscales for the present study and percentile scores relative to data norms of Gold-MSI [34].

Variable	Mean (SD) (Present Study)	Percentile Relative to Norms *
Active Engagement	31.33 (9.3)	17%
Perceptual Abilities	45.48 (8.4)	48%
Musical Training	21.74 (8.5)	47%
Singing Abilities	26.75 (8.4)	46%
Emotions	30.49 (5.6)	44%

* Müllensiefen et al. [34]. *N* = 147,633, mean age = 35.2 years (SD = 15).

## Data Availability

The data presented in this study are available on request from the corresponding author.
